# Adaptative Up-Regulation of PRX2 and PRX5 Expression Characterizes Brain from a Mouse Model of Chorea-Acanthocytosis

**DOI:** 10.3390/antiox11010076

**Published:** 2021-12-29

**Authors:** Enrica Federti, Alessandro Matte, Veronica Riccardi, Kevin Peikert, Seth L. Alper, Adrian Danek, Ruth H. Walker, Angela Siciliano, Iana Iatcenko, Andreas Hermann, Lucia De Franceschi

**Affiliations:** 1 Department of Medicine, University of Verona AOUI Verona, 37134 Verona, Italy; enrica.federti@univr.it (E.F.); alessandro.matte@univr.it (A.M.); veronica.riccardi@univr.it (V.R.); siciliano.angela@univr.it (A.S.); iana.iatcenko@univr.it (I.I.); 2 Translational Neurodegeneration Section “Albrecht-Kossel”, Department of Neurology, University Medical Center Rostock, University of Rostock, 18051 Rostock, Germany; kevin.peikert@med.uni-rostock.de (K.P.); andreas.hermann@med.uni-rostock.de (A.H.); 3 Beth Israel Deaconess Medical Center, Departments of Medicine, Harvard Medical School, Boston, MA 02215, USA; salper@bidmc.harvard.edu; 4 Department of Neurology, University Hospital, 80539 Munich, Germany; danek@lmu.de; 5 James J. Peters Veterans Affairs Medical Center, Department of Neurology, Bronx, NY 10468, USA; ruth.walker@mssm.edu; 6 Department of Neurology, Icahn School of Medicine at Mount Sinai, New York, NY 10029, USA; 7 Center for Transdisciplinary Neurosciences Rostock (CTNR), University Medical Center Rostock, University of Rostock, 18051 Rostock, Germany

**Keywords:** peroxiredoxin 2, peroxiredoxin 5, neuroinflammation, chorea-acanthocytosis, nilotinib, Lyn, oxidative stress

## Abstract

The peroxiredoxins (PRXs) constitute a ubiquitous antioxidant. Growing evidence in neurodegenerative disorders such as Parkinson’s disease (PD) or Alzheimer’s disease (AD) has highlighted a crucial role for PRXs against neuro-oxidation. Chorea-acanthocytosis/*Vps13A* disease (ChAc) is a devastating, life-shortening disorder characterized by acanthocytosis, neurodegeneration and abnormal proteostasis. We recently developed a *Vps13a^−/−^* ChAc-mouse model, showing acanthocytosis, neurodegeneration and neuroinflammation which could be restored by LYN inactivation. Here, we show in our *Vps13a^−/−^* mice protein oxidation, NRF2 activation and upregulation of downstream cytoprotective systems NQO1, SRXN1 and TRXR in basal ganglia. This was associated with upregulation of PRX2/5 expression compared to wild-type mice. PRX2 expression was age-dependent in both mouse strains, whereas only *Vps13a^−/−^* PRX5 expression was increased independent of age. LYN deficiency or nilotinib-mediated LYN inhibition improved autophagy in *Vps13a^−/−^* mice. In *Vps13a^−/−^; Lyn^−/−^* basal ganglia, absence of LYN resulted in reduced NRF2 activation and down-regulated expression of PRX2/5, SRXN1 and TRXR. Nilotinib treatment of *Vps13a^−/−^* mice reduced basal ganglia oxidation, and plasma PRX5 levels, suggesting plasma PRX5 as a possible ChAc biomarker. Our data support initiation of therapeutic Lyn inhibition as promptly as possible after ChAc diagnosis to minimize development of irreversible neuronal damage during otherwise inevitable ChAc progression.

## 1. Introduction

The peroxiredoxins (PRXs) constitute a ubiquitous antioxidant system encompassing typical two-cysteine (Cys) Prxs (1–4), a single atypical two-Cys Prx (PRX5) and a single one-Cys Prx (PRX6) [1,2,3,4,5,6,7]. PRX2 and PRX5 use two Cys residues to accomplish the task of detoxifying a vast range of organic peroxides, H_2_O_2_ and peroxynitrite. Hyperoxidation of the peroxidatic cysteine (Cys-S_P_O_2_H) leads to formation of catalytically inactive high molecular weight oligomers with chaperone function [2]. For this reason, two-Cys PRXs require both thioredoxin reductase (TRXR) and sulfiredoxin-1 (SRXN1) to be reduced and reactivated [5,8,9,10,11,12,13]. SRXN1, in particular, restores overoxidized PRXSO3 to the TRXR cycle, preventing inactivation of PRXs [14]. Since oxidation is crucial for cell homeostasis, PRXs are highly regulated by several modifications such as phosphorylation, acetylation, glutathionylation and nitrosylation [15,16,17]. 

Growing evidence in both cell and animal models of neurodegenerative disorders such as Parkinson disease (PD), Alzheimer disease (AD) and Huntington disease (HD) has highlighted a novel and crucial role of PRX2 in defense against oxidative stress. Such stress is often related to pathologic accumulation of neurotoxic proteins, including Aβ amyloid and tau-proteins [18,19,20,21]. PRX2 up-regulation has been documented in brains of patients with AD and with PD [19,22]. This finding is paralleled by increased PRX5 expression in models of AD and PD, acting in cells as both antioxidant and atypical chaperone, and outside cells as a modulator of local inflammatory response [19,23,24,25,26]. Plasma levels of PRX2 and PRX5 are also modulated in clinical settings such as autism or stroke, suggesting use of plasma PRX2 and/or PRX5 as possible biomarkers for neurodegeneration and/or neuroinflammation [26,27]. Indeed, accumulation of overoxidized PRXs (PRXSO3) requires up-regulation of SRXN1. SRXN1, in turn, is a downstream gene of NRF2, a redox-related transcription factor playing an important role in the pathogenesis of neurodegenerative disorders [28].

Chorea-acanthocytosis/*Vps13A* disease (ChAc) is a devastating disease of young adult causing blood cell acanthocytosis and a complex neurological syndrome with significantly reduced live span. ChAc presents clinically as a choreatic hypokinetic movement disorder, associated with progressive cognitive decline and peripheral neuro-(myo) pathy [29]. Nearly half of ChAc patients suffer from epilepsy, which is often refractory to therapy. ChAc is almost always caused by homozygous or compound heterozygous mutations in *VPS13A* (Vacuolar Protein Sorting 13 Homolog A) leading to lack of the encoded protein, Chorein [30,31,32]. *VPS13A* has one ancestor in yeast and is conserved across species. Proteins of the *VPS13A* gene family, including *VPS13A*/chorein, localize to membrane contact sites acting as bulk lipid transporters between the membranes of different organelles [33,34]. We have shown previously that the absence of *VPS13A*/chorein is associated with accumulation of active Lyn, a Src family kinase which represents a promising drug target for ChAc [29,35,36,37].

In mice genetically lacking *Vps13a* (*Vps13a^−/−^*), we found impaired autophagy in both erythroid cells and basal ganglia [29,35,36,37]. This was accompanied by accumulation of neurotoxic proteins such as phospho-Tau proteins, and by active Lyn stabilized in high molecular weight protein complexes with HSP90 [36,38]. We also documented increased microglia, and NF-kB p65 activation and up-regulation of pro-inflammatory cytokines such as IL-1β, indicating the presence of neuroinflammation in this mouse model of ChAc [29,35,36,37]. In both human erythroid cells and in neurons-derived from iPS cells from patients with ChAc, we have shown that phospho-Lyn accumulation and impairment of autophagy can be reduced by treatment with the Tyr-kinase inhibitor, dasatinib, used in clinical practice for oncohematologic disorders [29,35,36,37]. In single ChAc patients, dasatinib treatment was reasonable safe and showed target engagement at least in the erythroid cells, whereas treatment response in the central nervous system could neither be proven nor disproven [37]. We found that in vivo treatment of *Vps13a^−/−^* mice with nilotinib, a Tyr kinase inhibitor that can permeate the brain blood barrier (BBB), reduces accumulation of active Lyn, improves autophagy, and ameliorates neuroinflammation [36]. 

Here, we show that plasma levels of PRX2 and PRX5 are increased in both *Vps13a^−/−^* mice and in ChAC patients. To explore the source of plasma PRX2 and PRX5, we evaluated PRX2 and PRX5 in red cells. We found reduction in PRX2 expression in both cytoplasm and membrane fractions of erythrocytes from *Vps13a^−/−^* mice as compared to healthy erythrocytes. This finding agrees with our previous report in human ChAc erythrocytes [35,38], whereas PRX5 expression in wild-type and *Vps13a^−/−^* mouse red cells was similar. Analyses of *Vps13a^−/−^* mouse basal ganglia revealed increased protein oxidation and activation of the redox-related transcription factor, NRF2, associated with up-regulation of PRX2 and PRX5 expression and their reducing systems: thioredoxin-reductase (TRXR) and sulfiredoxin-1 (SRXN1). Basal ganglia from *Vps13a^−/−^* mice exhibited increased PRXSO3 levels, suggesting a relation between increased local oxidative stress and impaired autophagy [36]. We previously documented improvement of autophagy in double-knockout mice lacking both VPS13a and LYN [36]. In basal ganglia from *Vps13a^−/−^; Lyn^−/−^* mice, the absence of LYN results in reduction of NRF2 activation and down-regulated expression of PRX2/5, SRXN1 and TRXR as compared to *Vps13a^−/−^* mouse basal ganglia. We also observed reduction in PRXSO3, supporting the observation that improvement of autophagy is linked to reduction of cellular oxidative stress. As proof of concept, we administrated Nilotinb to *Vps13a^−/−^* mice. In basal ganglia from nilotinib-treated *Vps13a^−/−^* mice we found reduced NRF2 activation and down-regulated expression of PRX2/5, TRXR and SRXN1, paralleled by a decrease in PRXSO3. 

Our data collectively show for the first time that in *Vps13a^−/−^* mouse basal ganglia, PRX2/5 might represent neuroprotective systems against oxidation and neuroinflammation linked to impaired autophagy. Plasma PRX5 more than plasma PRX2 might reflect neurodegeneration related to the absence of VPS13a expression. Nilotinib improved autophagy, indirectly attenuated oxidation and reduced PRX2/PRX5 expression, supporting re-purposing of Lyn kinase inhibitors as a possible treatment for patients with ChAc. In addition, plasma PRX5 might represent a disease marker useful in follow-up of patients since plasma PRX5 is increased in *Vps13a^−/−^* mice and reduced by nilotinib treatment. 

## 2. Materials and Methods 

### 2.1. Mouse Strains and Design of the Study

12- and 18-month-old C57B6/2J wild-type (WT) mice, *Vps13a^−/−^* mice and *Vps13a^−/−^ Lyn^−/−^* mice were studied [36]. Whenever indicated, WT and *Vps13a^−/−^* mice were treated daily with vehicle (tap water) or nilotinib (25 mg/Kg/day). Nilotinib was administered by once daily gavage to WT or *Vps13a^−/−^* mice of ages 11 or 17 months for periods of either 3 or 6 months [36]. Isoflurane-anesthetized mice were euthanized and randomly assigned to experimental groups. Brains were acutely dissected to isolate basal ganglia (BG, consisting of corpus striatum), which was rapidly frozen in liquid nitrogen. Red cells and plasma collected from both *Vps13a^−/−^* mice and from ChAc patients were analyzed for levels of PRX2 and PRX5 by Western-blot analysis [37,39,40,41]. 

### 2.2. Patient Characteristics 

Plasma samples from ChAc patients described in two previous studies were analyzed [35,36,38]. In brief, diagnosis was based on clinical manifestations, the absence of chorein in Western blot analysis, and genetic testing. All patients gave their informed consent for sample preparation and publication of the data. Patients and healthy control blood donors were enrolled in ongoing studies on the pathogenesis and natural history of neurodegenerative diseases approved by the institutional review board of the Technische Universität Dresden, Germany (EK 45022009, EK 78022015) and Comitato Etico Verona e Rovigo (FGFITA3).

### 2.3. Western-Blot Analysis and Immunoprecipitation Assay

Plasma, red cell membrane (ghost) and cytosol fractions obtained as previously reported [37,39,40,41] were analyzed by SDS-PAGE. Basal ganglia were homogenized, lysed and analyzed by SDS-PAGE [36]. Details are reported in Supplementary Materials. Gels were transferred to nitrocellulose membranes for immunoblot analysis with specific antibodies reported in Supplementary Methods. Oxidized proteins were monitored using OxyBlot Protein Oxidation Detection Kit (EMD Millipore) as previously reported [42,43]. In brief, carbonylated proteins were detected by reacting with 2,4-dinitrophenylhydrazine (DNPH) and blotted with anti-dinitrophenyl antibody [42,43]. 

### 2.4. Statistical Analysis 

Data were analyzed using either t-test or one-way ANOVA for multiple comparisons. A difference with a *p* 0.05 was considered significant. 

## 3. Results

### 3.1. Plasma PRX2 and PRX5 Are Increased in Both Vps13a^−/−^ Mice and Patients with ChAc

**Figure 1:**
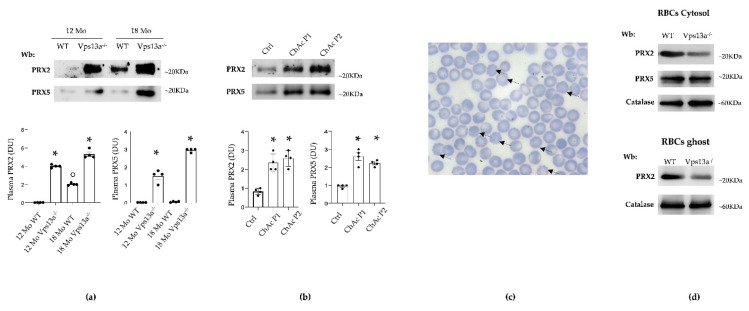
(**a**) Western blot analysis (Wb) of plasma from WT and *Vps13a^−/−^* mice at 12 and 18 months (Mo) of age, using specific antibodies against PRX2 and PRX5. One representative gel from 4 with similar results is shown. Densitometric analysis is shown in lower panel. Data are presented as means ± SEM, *n* = 4 * *p* 0.05 compared to WT; ° *p* 0.05 compared to 12 months old WT mice. Loading controls are shown in Figure S1a (**b**) Western-blot (Wb) analysis of PRX2 and PRX5 in plasma samples from healthy control (Ctrl), ChAc patient 1 (P1) and ChAc patient 2 (P2), corresponding to ChAc patients reported in De Franceschi L et al. [36]. Densitometric analysis is shown in lower panel. Data are presented as means ± SEM, *n* = 4 * *p* 0.05 compared to healthy controls. Loading controls are shown in Figure S1b. (**c**) Morphologic analysis of peripheral blood from *Vps13a^−/−^* mice. Blood smears stained with May Grunwald Giemsa. Cells were imaged under oil at 100× magnification using Panfluor objective 1.30 numeric aperture on a Nikon Eclipse DS-5 M camera and processed with Digital Slide (DS-L1) Nikon. Black arrows indicate acanthocytes in *Vps13a^−/−^* mice. (**d**) Western-blot (Wb) analysis of PRX2 and PRX5 in cytosolic fraction (upper panel) and of PRX2 in membrane fraction (ghost, lower panel) from red cells (RBC) of WT and *Vps13a^−/−^* mice at 12 months of age. Catalase was used as protein loading control. The blot shown is representative of four with similar results. Densitometric analyses are presented in Supplementary Figure S1c.

We previously reported that 12- and 18-month-old *Vps13a^−/−^* mice recapitulate the clinical and biologic phenotypes of ChAc [36]. As shown in Figure 1a, plasma levels of PRX2 and PRX5 were significantly higher in *Vps13a^−/−^* mice aged 12 and 18 months than in corresponding wild-type animals. PRX5 increased with age in *Vps13a^−/−^* mice but not in wild-type mice (Figure 1a and Figure S1a). Plasma PRX2 levels were significantly higher in 18-month-old wild-type mice than in younger healthy animals (Figure 1a and Figure S1a). Levels of PRX2 and PRX5 in plasma from ChAc patients was significantly higher than in plasma of healthy subjects (Figure 1b and Figure S1b). Since the normal range of circulating PRXs remains undefined, we have assumed that plasma PRX concentrations in wild-type animals and in human healthy controls are not pathologic [44]. 

As PRX2 and PRX5 are expressed in red cells [8,10,45], we analyzed PRX2 and PRX5 in cytosol fractions from erythrocytes of wild-type and erythrocytes (including circulating acanthocytes, Figure 1c) of *Vps13a^−/−^* mice (Figure 1d, upper panel). PRX2 expression in *Vps13a^−/−^* mouse erythrocytes was significantly lower than in wild-type red cells, whereas PRX5 expression was similar in both mouse strains (Figure 1d, upper panel; Figure S1c). In agreement with our previous report in human ChAc red cells [38], the amount of PRX2 translocated to the membrane (ghost fraction) in *Vps13a^−/−^* red cells was significantly lower than in red cell membrane fractions from wild-type animals (Figure 1d, lower panel; Figure S1c). This difference is related to the perturbation of multiprotein complexes involving band 3, the docking site for PRX2 [8].

Since PRX2 and PRX5 might be released into plasma by additional cell types besides red cells, the increased plasma levels of PRX2 and PRX5 observed in both *Vps13a^−/−^* mice and human ChAc patients also reflect the neurodegeneration and neuroinflammation observed in ChAc. 

### 3.2. Activation of NRF2 and Up-Regulation of PRX2/5 Characterizes Basal Ganglia from Vps13a^−/−^ Mice

**Figure 2:**
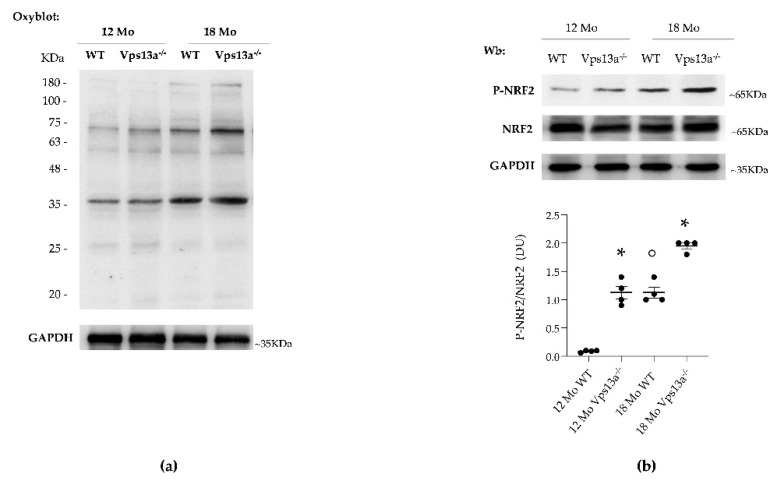
(**a**) Basal ganglia soluble fractions from WT and *Vps13a^−/−^* mice aged 12 and 18 months (Mo) of were analyzed on 11% SDS-PAGE and subjected to OxyBlot. Carbonylated proteins (1 mg) were detected by treating with 2,4-dinitrophenylhydrazine and blotted with anti-DNP antibody. Representative of 4 blots with similar results. Quantitation of band area is reported in Supplementary Figure S2a. (**b**) Western-blot (Wb) analysis of phospho-NRF2 and total NRF2 in isolated basal ganglia of wild-type (WT) and *Vps13a^−/−^* mice aged 12 and 18 months. GADPH was used as loading control. Representative of 4 blots with similar results. Densitometric analysis is shown in lower panel. Data are presented as means±SEM, *n* = 4 * *p* 0.05 compared to WT animals; ° *p* 0.05 compared to 12-month-old WT mice.

NRF2 is a key redox-related transcription factor, linked to PRXs expression and function [11]. Studies of neurodegenerative disorders such as PD or AD have shown oxidation to be an early neurotoxicity marker of abnormal proteostasis [46,47,48,49,50,51,52,53]. We observed age-dependent increases in protein oxidation in basal ganglia isolated from *Vps13a^−/−^* and wild-type mice of ages 12 and 18 months, as determined by OxyBlot (Figure 2a and Figure S2). This increase was greater in basal ganglia from *Vps13^−/−^* mice than in wild-type basal ganglia. We also found age-dependent NRF2 activation in basal ganglia isolated from *Vps13a^−/−^* and wild-type mice of ages 12 and 18 months (Figure 2b), greater in the former than the latter (Figure 2b).

**Figure 3:**
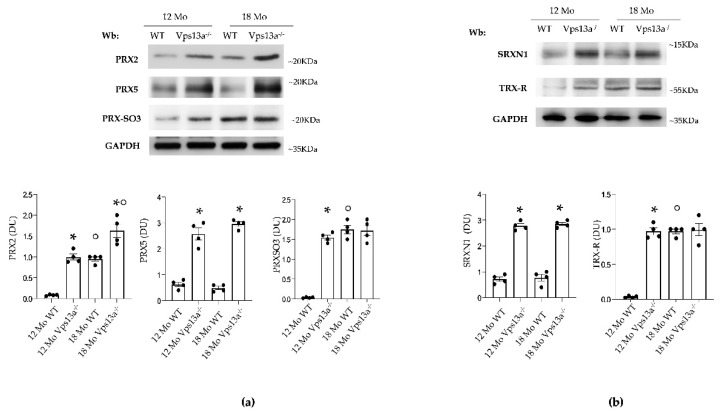
(**a**) Western-blot (Wb) analysis of PRX2, PRX5 and PRX-SO3 in isolated basal ganglia from wild-type (WT) and *Vps13a^−/−^* mice aged 12 and 18 months (Mo). One representative immunoblot of four with similar results is shown. GADPH was used as loading control. Densitometric analysis is shown in lower panel. Data are shown as means ± SEM, *n* = 4; * *p* 0.05 compared to WT animals; ° *p* 0.05 compared to 12-month-old WT mice. (**b**) Western-blot (Wb) analysis of sulfiredoxin 1 (SRXN1) and thioredoxin reductase (TRXR) in isolated basal ganglia from wild-type (WT) and *Vps13a^−/−^* mice aged 12 and 18 months). GADPH was used as loading control. Representative of four blots with similar results. Densitometric analysis is shown in lower panel. Data are shown as means ±SEM, *n* = 4; * *p* 0.05 compared to WT animals; ° *p* 0.05 compared to 12 months old WT mice.

Expression of PRX2 and PRX5 was significantly higher in basal ganglia from *Vps13a^−/−^* mice aged 12 and 18 months than in basal ganglia from corresponding wild-type animals (Figure 3a). This observation was associated with PRX-SO3 accumulation in basal ganglia from 12-month-old *Vps13a^−/−^* mice as compared to age-matched wild-type animals (Figure 3a). No major difference in PRX-SO3 was evident between basal ganglia from *Vps13a^−/−^* and wild type mice aged 18 months (Figure 3a). Similarly, we found up-regulation of SRXN1 expression in basal ganglia from *Vps13a^−/−^* mice aged 12 and 18 months as compared to corresponding wild-type animals (Figure 3b). TRXR expression was significantly higher in basal ganglia from 12-month-old *Vps13a^−/−^* mice than in wild-type animals of the same age. TRXR expression in basal ganglia did not significantly differ between 18-month-old mice of the two strains (Figure 3b).

Taken together our data indicate that oxidative stress is higher in basal ganglia from *Vps13a^−/−^* mice than in wild-type animals [35,36]. Chronic oxidative stress in basal ganglia from *Vps13a^−/−^* mice is associated with activation of NRF2 and up-regulation of both PRX2 and PRX5 cytoprotective systems. Although PRX2 expression is modulated by aging in both mouse strains, the increased age-independent expression of PRX5 and SRXN1 in *Vps13a^−/−^* mice appears related to the neurologic phenotype.

### 3.3. Improvement of Autophagy by Inhibition of Active Lyn Prevents NRF2 Activation and Down-Regulates PRX2/5 Expression in Vps13a^−/−^ Basal Ganglia

**Figure 4:**
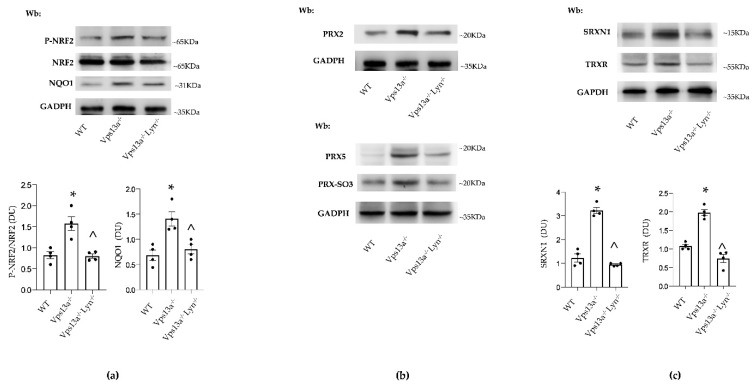
(**a**) Western blot (Wb) analysis of phospho-NRF2, total NRF2 and NQO1 in isolated basal ganglia from wild-type (WT), *Vps13a^−/−^* mice and *Vps13a^−/−^; Lyn^−/−^* mice aged 12 months. GADPH was used as protein loading control. Representative of 4 blots with similar results. Densitometric analysis is shown in lower panel. Data are shown as means±SEM, *n* = 4; * *p* 0.05 compared to WT animals; ^ *p* 0.05 compared to 12-month-old WT mice. (**b**) Western-blot (Wb) analysis of PRX2 (upper panel) and PRX5 and PRX-SO3 (lower panel) in isolated basal ganglia from wild-type (WT), *Vps13a^−/−^* mice and *Vps13a^−/−^; Lyn^−/−^* mice aged 12 months. GADPH was used as protein loading control. Representative of 4 blots with similar results. Densitometric analysis is shown in Supplementary Figure S3. (**c**) Western-blot (Wb) analysis of sulfiredoxin 1 (SRXN1) and thioredoxin reductase (TRXR) in isolated basal ganglia from wild-type (WT), *Vps13a^−/−^* mice and *Vps13a^−/−^; Lyn^−/−^* mice aged 12 months. GADPH was used as loading control. Representative of 4 blots with similar results. Densitometric analysis is shown in lower panel. Data are shown as means ± SEM, *n* = 4; * *p* 0.05 compared to WT animals; ^ *p* 0.05 compared to 12-month-old WT mice.

We previously reported that impaired autophagy in basal ganglia of *Vps13a^−/−^* mice results in accumulation of neurotoxic proteins and of active Lyn [35,36]. The genetic absence of Lyn (in *Vps13a^−/−^; Lyn^−/−^* mice) or therapeutic inhibition of active Lyn by nilotinib improves autophagy and beneficially impacts neuroinflammation in *Vps13a^−/−^* mice. We therefore evaluated NRF2 activity and PRX2/5 expression in *Vps13a^−/−^; Lyn^−/−^* mice. As shown in Figure 4a, we found that basal ganglia NRF2 activation was lower in *Vps13a^−/−^; Lyn^−/−^* mice than in *Vps13a^−/−^* animals. In agreement, we observed down-regulation of NQO1, a NRF2-regulated antioxidant system [11]. Expression of PRX2 and PRX5 in basal ganglia of *Vps13a^−/−^; Lyn^−/−^* mice was significantly lower than in *Vps13a^−/−^* animals (Figure 4b and Figure S3). Reduced accumulation of overoxidized PRXs (PRX-SO3) was also detected in basal ganglia of *Vps13a^−/−^; Lyn^−/−^* mice (Figure 4b and Figure S3). This was associated with lower basal ganglia expression of both SRXN1 and TRXR in *Vps13a^−/−^; Lyn^−/−^* than in *Vps13a^−/−^* animals (Figure 4c).

These data indicate that improvement of autophagy by genetic inactivation of LYN prevents activation of the NRF2 pathway, resulting in down-regulation of antioxidant systems such as NQO1, PRX2 and PRX5.

**Figure 5:**
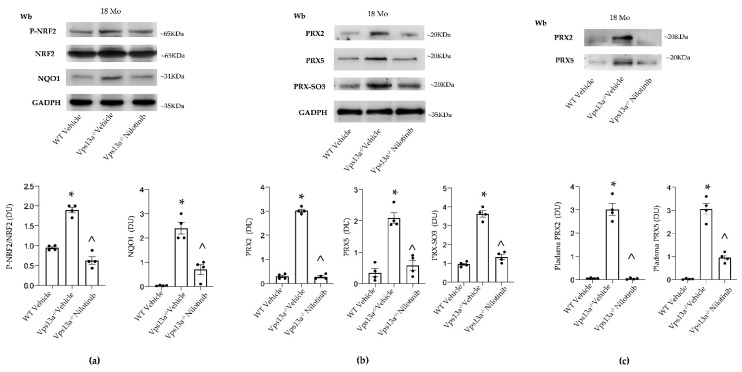
(**a**) Western-blot (Wb) analysis of phospho-NRF2, total NRF2 and NQO1 in isolated basal ganglia of wild-type (WT) and *Vps13a^−/−^* mice treated with either vehicle or nilotinib (25 mg/kg/day 6 months) at 18 months (Mo) of age. GADPH was used as loading control. Representative of 4 blots with similar results. Densitometric analysis is shown in lower panel. Data are shown as means ± SEM, *n* = 4; * *p* 0.05 compared to WT animals; ^ *p* 0.05 compared to vehicle treated *Vps13a^−/−^* mice. (**b**) Western-blot (Wb) analysis of PRX2, PRX5 and PRX-SO3 in isolated basal ganglia of wild-type (WT) and *Vps13a^−/−^* mice treated with either vehicle or nilotinib (25 mg/kg/day for 6 months) at 18 months of age. GADPH was used as protein loading control. Representative of 4 blots with similar results. Densitometric analysis is shown in lower panel. Data are shown as means ± SEM, *n* = 4; * *p* 0.05 compared to WT animals; ^ *p* 0.05 compared to vehicle treated *Vps13a^−/−^* mice. (**c**) Western-blot analysis (Wb), using specific antibodies against PRX2 and PRX5, in plasma from WT and *Vps13a^−/−^* mice treated with either vehicle or nilotinib (25 mg/kg/day for 6 months) at 18 months of age. Representative of 4 blots with similar results. Densitometric analysis is shown in lower panel. Data are shown as means ± SEM, *n* = 4; * *p* 0.05 compared to WT animals; ^ *p* 0.05 compared to vehicle treated *Vps13a^−/−^* mice.

As proof of concept, we treated *Vps13a^−/−^* mice with nilotinib, a specific LYN inhibitor that can permeate the BBB [36]. As shown in Figure 5a and Supplementary Figure S5a), nilotinib significantly reduced NRF2 activation in *Vps13a^−/−^* mice aged 12 and 18 months as compared to vehicle-treated animals. This result is consistent with down-regulation of the NRF2 dependent NQO1 antioxidant system and the reduction in protein oxidation observed in basal ganglia of nilotinib-treated *Vps13a^−/−^* mice aged 12 and 18 months as compared to vehicle-treated animals (Figure 5a, Supplementary Figure S4).

Basal ganglia expression of PRX2 and PRX5 was significantly lower in nilotinib-treated than in vehicle-treated *Vps13a^−/−^* mice aged 12 and 18 months and was associated with reduced accumulation of overoxidized PRXs (PRX-SO3) (Figure 5b and Supplementary Figure S5b). Most notably, plasma PRX5 levels in mice treated with nilotinib were lower than in vehicle-treated animals (Figure 5c and Figure S6).

## 4. Discussion

Our data indicate that oxidation is an early event in the pathogenesis of ChAc in *Vps13a^−/−^* mice, resembling other neurodegenerative disorders such as PD or AD [18,51,52,54]. Indeed, protein oxidation was detectable in basal ganglia from 12-month-old *Vps13a^−/−^* mice, whereas neuronal loss appeared not earlier than in 18 months old *Vps13a^−/−^* mice. These changes were absent from younger *Vps13a^−/−^* mice and from age-matched wild-type animals [36].

**Figure 6:**
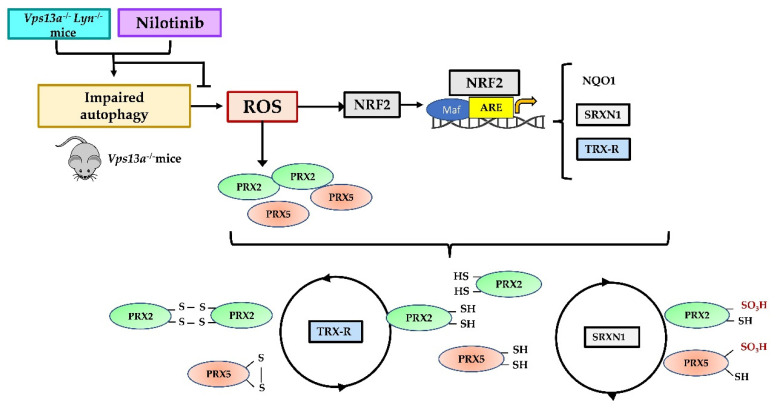
Schematic diagram. *Vps13a^−/−^* mice are characterized by impaired autophagy and neuroinflammation, with increased production of reactive oxygen species (ROS). Therapeutic activation of redox-related transcriptional factor NRF2 binds to antioxidant-responsive elements (ARE) of specific genes, promoting transcription of antioxidant proteins such as NQO1, SRXN1 and TRXR, in order to counteract producction of ROS and other reactive species. TRXR is required to reduce and reactivate PRX2 and PRX5. In highly oxidized environments, Prxs can be overoxidized (PRX-SO3). SRXN1 can restore overoxidized Prxs to their functional reduced state. These antioxidant proteins together orchestrate preservation of a beneficial equilibrium between antioxidants and oxidants. In double knockout *Vps13a^−/−^; Lyn^−/−^* mice and in *Vps13a^−/−^* animals treated with nilotinib, amelioration of authophagy beneficially affects oxidation, resulting in reduced ROS production.

In *Vps13a^−/−^* mice prolonged oxidation is associated with activation of NRF2 (pNRF2) and up-regulation of PRX2, PRX5 and SRXN1. pNRF2 levels increased with age in both WT and mutant mouse strains but were higher in *Vps13a^−/−^* mice. This was paralleled by up-regulation of PRX2 and TRXR. In contrast, expression of PRX5 and SRXN1 was increased in *Vps13a^−/−^* basal ganglia in an age-independent manner. PRX5 might thus be a more reliable marker of neuro-oxidative damage than PRX2 in *Vps13a^−/−^* mice. A possible anti-inflammatory activity of PRX5 might also be suggested in *Vps13a^−/−^* mice, based on previous reports in models of AD and PD [18]. The up-regulation of SRXN1 further emphasizes the severity of oxidation in basal ganglia from *Vps13a^−/−^* mice (Figure 6). SRXN1 restores PRXSO3 to the TRXR cycle, preventing overoxidation and inactivation of PRXs [28,53,55]. Indeed, both *Srxn1* and *TrxR* have ARE-elements under NRF2 regulation, indicating their importance in resistance against neuro-oxidation (Figure 6) [28]. Moreover, a neuroprotective role for SRXN1 has been previously suggested in in vitro models of neuronal cells exposed to oxidation [56,57].

To investigate the possibility that potentiation of cytoprotective systems can counteract oxidation in neurodegenerative disorders, different therapeutic strategies to restore redox balance have been tested in both cell and animal models of AD, PD and multiple sclerosis (MS) [51,52]. These strategies are based largely on pure antioxidants (such as N-acetylcysteine and ω3-fatty acid supplementation), or antioxidant/potentiators of NRF2 (such as quercitin and myricytin), or NRF2 antagonists (e.g., sulphoraphane and dimethylfumarate) [51,52,58]. Although beneficial antioxidant effects of these molecules have been reported in models for neurodegenerative disorders, the time of treatment initiation during the course of disease progression seems to be an important determinant of treatment impact.

We approached this dilemma by targeting autophagy to prevent accumulation of neurotoxic proteins. We took advantage of our previous studies showing amelioration of autophagy in double knockout *Vps13a^−/−^; Lyn^−/−^* mice as well as in *Vps13a^−/−^* animals treated with nilotinib [35,36]. In basal ganglia from *Vps13a^−/−^*; *Lyn^−/−^* mice, the absence of LYN activity prevented NRF2 activation and up-regulation of NRF2-related cytoprotective systems, including NQO1, SRX1 or TRXR. These effects were associated with down-regulation of PRX2 and PRX5 and decrease of PrxSO3, supporting the strategy of improving autophagy to limit oxidation. As proof of concept, we found reduced protein oxidation and NRF2 activation in basal ganglia from *Vps13a^−/−^* mice treated with nilotinb (Figure 6), a drug previously tested in patients with PD, dementia with Lewy Bodies and AD in different clinical studies [59,60,61,62,63,64]. A major limitation of these human studies is late initiation of treatment long after onset and progression of PD. Thus, initiation of treatment(s) directly targeting oxidation (e.g., antioxidant) or reducing oxidation by improvement of autophagy (e.g., Lyn inhibitors) must begin as early as possible in the disease course to prevent irreversible cellular damage. Indeed, PRX5 levels are reduced in nilotinib-treated *Vps13a^−/−^* mice and appear to be more specific than PRX2 levels as indicators of neuro-oxidation and neuroinflammation, whereas plasma PRX5 levels might serve to guide clinical decision-making in human ChAc. Consistent with this observation, the modulation of plasma PRX5 levels in rodents and humans after brain damage [23,26,65,66] supports plasma PRX5 as both a marker of neurologic damage and a possible therapeutic target.

In conclusion, our study highlights the novel role of oxidation as a contributor to the pathogenesis of ChAc in mice genetically lacking the *Vps13* gene product, chorein. In *Vps13a^−/−^* animals, activation of Nrf2 and related up-regulation of antioxidant systems is inadequate in the context of the prolonged oxidation linked to the impaired autophagy that characterizes ChAc. Since pathological protein oxidation is related to accumulation of neurotoxic proteins, therapeutic strategies to improve protein clearance represents an interesting therapeutic approach to ChAc. PRX5 might thus act both in cytoprotective and anti-inflammatory manners. Our observation that the increased basal ganglia expression and plasma levels of PRX5 in *Vps13a^−/−^* mice are reduced by nilotinib treatment lends support to the use of plasma PRX5 levels as disease biomarker possibly transferable to human studies. Collectively, our data further support the initiation of therapeutic Lyn inhibition as soon as possible after diagnosis of ChAc, to produce the highest likelihood of preventing irreversible cellular damage related to the natural history of the disease.

## Data Availability

Data is contained within the article or Supplementary Material.

## Supplementary Materials

The following are available online at https://www.mdpi.com/article/10.3390/antiox11010076/s1, Supplementary Methods, Figures S1–S6: Original images for blots.

## References

[1] Wood Z.A., Schröder E., Harris J.R., Poole L.B. (2003). Structure, mechanism and regulation of peroxiredoxins. Trends Biochem. Sci..

[2] Wood Z.A., Poole L.B., Hantgan R.R., Karplus P.A. (2002). Dimers to doughnuts: Redox-sensitive oligomerization of 2-cysteine peroxiredoxins. Biochemistry.

[3] Low F.M., Hampton M.B., Winterbourn C.C. (2008). Peroxiredoxin 2 and Peroxide Metabolism in the Erythrocyte. Antioxid. Redox Signal..

[4] Manta B., Hugo M., Ortiz C., Ferrer-Sueta G., Trujillo M., Denicola A. (2009). The peroxidase and peroxynitrite reductase activity of human erythrocyte peroxiredoxin 2. Arch. Biochem. Biophys..

[5] Rhee S.G. (2016). Overview on Peroxiredoxin. Mol. Cells.

[6] Rhee S.G., Kang S.W., Chang T.S., Jeong W., Kim K. (2001). Peroxiredoxin, a novel family of peroxidases. IUBMB Life.

[7] Chae H.Z., Uhm T.B., Rhee S.G. (1994). Dimerization of thiol-specific antioxidant and the essential role of cysteine 47. Proc. Natl. Acad. Sci. USA.

[8] Matte A., Pantaleo A., Ferru E., Turrini F., Bertoldi M., Lupo F., Siciliano A., Ho Zoon C., De Franceschi L. (2014). The novel role of peroxiredoxin-2 in red cell membrane protein homeostasis and senescence. Free Radic. Biol. Med..

[9] Matte A., Bertoldi M., Mohandas N., An X., Bugatti A., Brunati A.M., Rusnati M., Tibaldi E., Siciliano A., Turrini F. (2013). Membrane association of peroxiredoxin-2 in red cells is mediated by the N-terminal cytoplasmic domain of band 3. Free Radic. Biol. Med..

[10] De Franceschi L., Bertoldi M., Matte A., Santos Franco S., Pantaleo A., Ferru E., Turrini F. (2013). Oxidative stress and beta-thalassemic erythroid cells behind the molecular defect. Oxid. Med. Cell. Longev..

[11] Matte A., De Falco L., Iolascon A., Mohandas N., An X., Siciliano A., Leboeuf C., Janin A., Bruno M., Choi S.Y. (2015). The Interplay Between Peroxiredoxin-2 and Nuclear Factor-Erythroid 2 Is Important in Limiting Oxidative Mediated Dysfunction in beta-Thalassemic Erythropoiesis. Antioxid. Redox Signal..

[12] Matte A., De Falco L., Federti E., Cozzi A., Iolascon A., Levi S., Mohandas N., Zamo A., Bruno M., Lebouef C. (2018). Peroxiredoxin-2: A Novel Regulator of Iron Homeostasis in Ineffective Erythropoiesis. Antioxid. Redox Signal..

[13] Rhee S.G., Chae H.Z., Kim K. (2005). Peroxiredoxins: A historical overview and speculative preview of novel mechanisms and emerging concepts in cell signaling. Free Radic. Biol. Med..

[14] Woo H.A., Jeong W., Chang T.S., Park K.J., Park S.J., Yang J.S., Rhee S.G. (2005). Reduction of cysteine sulfinic acid by sulfiredoxin is specific to 2-cys peroxiredoxins. J. Biol. Chem..

[15] Hall A., Nelson K., Poole L.B., Karplus P.A. (2011). Structure-based insights into the catalytic power and conformational dexterity of peroxiredoxins. Antioxid. Redox Signal..

[16] Rhee S.G., Woo H.A. (2020). Multiple functions of 2-Cys peroxiredoxins, I and II, and their regulations via post-translational modifications. Free Radic. Biol. Med..

[17] Romero-Puertas M.C., Laxa M., Matte A., Zaninotto F., Finkemeier I., Jones A.M., Perazzolli M., Vandelle E., Dietz K.J., Delledonne M. (2007). S-nitrosylation of peroxiredoxin II E promotes peroxynitrite-mediated tyrosine nitration. Plant. Cell.

[18] Szeliga M. (2020). Peroxiredoxins in Neurodegenerative Diseases. Antioxidants.

[19] Stepler K.E., Mahoney E.R., Kofler J., Hohman T.J., Lopez O.L., Robinson R.A.S. (2020). Inclusion of African American/Black adults in a pilot brain proteomics study of Alzheimer’s disease. Neurobiol. Dis..

[20] Fang J., Nakamura T., Cho D.H., Gu Z., Lipton S.A. (2007). S-nitrosylation of peroxiredoxin 2 promotes oxidative stress-induced neuronal cell death in Parkinson’s disease. Proc. Natl. Acad. Sci. USA.

[21] Sorolla M.A., Reverter-Branchat G., Tamarit J., Ferrer I., Ros J., Cabiscol E. (2008). Proteomic and oxidative stress analysis in human brain samples of Huntington disease. Free Radic. Biol. Med..

[22] Basso M., Giraudo S., Corpillo D., Bergamasco B., Lopiano L., Fasano M. (2004). Proteome analysis of human substantia nigra in Parkinson’s disease. Proteomics.

[23] Shichita T., Hasegawa E., Kimura A., Morita R., Sakaguchi R., Takada I., Sekiya T., Ooboshi H., Kitazono T., Yanagawa T. (2012). Peroxiredoxin family proteins are key initiators of post-ischemic inflammation in the brain. Nat. Med..

[24] Jin M.H., Lee Y.H., Kim J.M., Sun H.N., Moon E.Y., Shong M.H., Kim S.U., Lee S.H., Lee T.H., Yu D.Y. (2005). Characterization of neural cell types expressing peroxiredoxins in mouse brain. Neurosci. Lett..

[25] Kim B., Park J., Chang K.T., Lee D.S. (2016). Peroxiredoxin 5 prevents amyloid-beta oligomer-induced neuronal cell death by inhibiting ERK-Drp1-mediated mitochondrial fragmentation. Free Radic. Biol. Med..

[26] Kunze A., Zierath D., Tanzi P., Cain K., Becker K. (2014). Peroxiredoxin 5 (PRX5) is correlated inversely to systemic markers of inflammation in acute stroke. Stroke.

[27] Abruzzo P.M., Matte A., Bolotta A., Federti E., Ghezzo A., Guarnieri T., Marini M., Posar A., Siciliano A., De Franceschi L. (2019). Plasma peroxiredoxin changes and inflammatory cytokines support the involvement of neuro-inflammation and oxidative stress in Autism Spectrum Disorder. J. Transl. Med..

[28] Wu J., Chen Y., Yu S., Li L., Zhao X., Li Q., Zhao J., Zhao Y. (2017). Neuroprotective effects of sulfiredoxin-1 during cerebral ischemia/reperfusion oxidative stress injury in rats. Brain Res. Bull..

[29] Peikert K., Danek A., Hermann A. (2018). Current state of knowledge in Chorea-Acanthocytosis as core Neuroacanthocytosis syndrome. Eur. J. Med. Genet..

[30] Dobson-Stone C., Danek A., Rampoldi L., Hardie R.J., Chalmers R.M., Wood N.W., Bohlega S., Dotti M.T., Federico A., Shizuka M. (2002). Mutational spectrum of the CHAC gene in patients with chorea-acanthocytosis. Eur. J. Hum. Genet..

[31] Rampoldi L., Dobson-Stone C., Rubio J.P., Danek A., Chalmers R.M., Wood N.W., Verellen C., Ferrer X., Malandrini A., Fabrizi G.M. (2001). A conserved sorting-associated protein is mutant in chorea-acanthocytosis. Nat. Genet..

[32] Ueno S., Maruki Y., Nakamura M., Tomemori Y., Kamae K., Tanabe H., Yamashita Y., Matsuda S., Kaneko S., Sano A. (2001). The gene encoding a newly discovered protein, chorein, is mutated in chorea-acanthocytosis. Nat. Genet..

[33] Leonzino M., Reinisch K.M., De Camilli P. (2021). Insights into VPS13 properties and function reveal a new mechanism of eukaryotic lipid transport. Biochim. Biophys. Acta Mol. Cell Biol. Lipids.

[34] Dziurdzik S.K., Conibear E. (2021). The Vps13 Family of Lipid Transporters and Its Role at Membrane Contact Sites. Int. J. Mol. Sci..

[35] Lupo F., Tibaldi E., Matte A., Sharma A.K., Brunati A.M., Alper S.L., Zancanaro C., Benati D., Siciliano A., Bertoldi M. (2016). A new molecular link between defective autophagy and erythroid abnormalities in chorea-acanthocytosis. Blood.

[36] Peikert K., Federti E., Matte A., Constantin G., Pietronigro E.C., Fabene P.F., Defilippi P., Turco E., Del Gallo F., Pucci P. (2021). Therapeutic targeting of Lyn kinase to treat chorea-acanthocytosis. Acta Neuropathol. Commun..

[37] Peikert K., Glass H., Federti E., Matte A., Pelzl L., Akgun K., Ziemssen T., Ordemann R., Lang F., Patients T. (2021). Targeting Lyn Kinase in Chorea-Acanthocytosis: A Translational Treatment Approach in a Rare Disease. J. Pers. Med..

[38] De Franceschi L., Tomelleri C., Matte A., Brunati A.M., Bovee-Geurts P.H., Bertoldi M., Lasonder E., Tibaldi E., Danek A., Walker R.H. (2011). Erythrocyte membrane changes of chorea-acanthocytosis are the result of altered Lyn kinase activity. Blood.

[39] De Franceschi L., Olivieri O., Miraglia del Giudice E., Perrotta S., Sabato V., Corrocher R., Iolascon A. (1997). Membrane cation and anion transport activities in erythrocytes of hereditary spherocytosis: Effects of different membrane protein defects. Am. J. Hematol..

[40] Brugnara C., de Franceschi L. (1993). Effect of cell age and phenylhydrazine on the cation transport properties of rabbit erythrocytes. J. Cell Physiol..

[41] Kalish B.T., Matte A., Andolfo I., Iolascon A., Weinberg O., Ghigo A., Cimino J., Siciliano A., Hirsch E., Federti E. (2015). Dietary omega-3 fatty acids protect against vasculopathy in a transgenic mouse model of sickle cell disease. Haematologica.

[42] Matte A., Federti E., Kung C., Kosinski P.A., Narayanaswamy R., Russo R., Federico G., Carlomagno F., Desbats M.A., Salviati L. (2021). The pyruvate kinase activator mitapivat reduces hemolysis and improves anemia in a beta-thalassemia mouse model. J. Clin. Investig..

[43] Matte A., Lupo F., Tibaldi E., Di Paolo M.L., Federti E., Carpentieri A., Pucci P., Brunati A.M., Cesaro L., Turrini F. (2020). Fyn specifically regulates the activity of red cell glucose-6-phosphate-dehydrogenase. Redox Biol..

[44] El Eter E., Al Masri A., Habib S., Al Zamil H., Al Hersi A., Al Hussein F., Al Omran M. (2014). Novel links among peroxiredoxins, endothelial dysfunction, and severity of atherosclerosis in type 2 diabetic patients with peripheral atherosclerotic disease. Cell Stress Chaperones.

[45] Pantaleo A., Ferru E., Pau M.C., Khadjavi A., Mandili G., Matte A., Spano A., De Franceschi L., Pippia P., Turrini F. (2016). Band 3 Erythrocyte Membrane Protein Acts as Redox Stress Sensor Leading to Its Phosphorylation by p (72) Syk. Oxid. Med. Cell. Longev..

[46] Jazvinscak Jembrek M., Orsolic N., Mandic L., Sadzak A., Segota S. (2021). Anti-Oxidative, Anti-Inflammatory and Anti-Apoptotic Effects of Flavonols: Targeting Nrf2, NF-kappaB and p53 Pathways in Neurodegeneration. Antioxidants.

[47] Bolduc J., Koruza K., Luo T., Malo Pueyo J., Vo T.N., Ezerina D., Messens J. (2021). Peroxiredoxins wear many hats: Factors that fashion their peroxide sensing personalities. Redox Biol..

[48] Bi M., Du X., Xiao X., Dai Y., Jiao Q., Chen X., Zhang L., Jiang H. (2021). Deficient immunoproteasome assembly drives gain of alpha-synuclein pathology in Parkinson’s disease. Redox Biol..

[49] Al-Mubarak B.R., Bell K.F.S., Chowdhry S., Meakin P.J., Baxter P.S., McKay S., Dando O., Ashford M.L.J., Gazaryan I., Hayes J.D. (2021). Non-canonical Keap1-independent activation of Nrf2 in astrocytes by mild oxidative stress. Redox Biol..

[50] Villavicencio Tejo F., Quintanilla R.A. (2021). Contribution of the Nrf2 Pathway on Oxidative Damage and Mitochondrial Failure in Parkinson and Alzheimer’s Disease. Antioxidants.

[51] Davies D.A., Adlimoghaddam A., Albensi B.C. (2021). Role of Nrf2 in Synaptic Plasticity and Memory in Alzheimer’s Disease. Cells.

[52] Brandes M.S., Gray N.E. (2020). NRF2 as a Therapeutic Target in Neurodegenerative Diseases. ASN Neuro.

[53] Johnson D.A., Johnson J.A. (2015). Nrf2--a therapeutic target for the treatment of neurodegenerative diseases. Free Radic. Biol. Med..

[54] Zhu H., Santo A., Li Y. (2012). The antioxidant enzyme peroxiredoxin and its protective role in neurological disorders. Exp. Biol. Med..

[55] Zhang M., An C., Gao Y., Leak R.K., Chen J., Zhang F. (2013). Emerging roles of Nrf2 and phase II antioxidant enzymes in neuroprotection. Prog. Neurobiol..

[56] Li Q., Yu S., Wu J., Zou Y., Zhao Y. (2013). Sulfiredoxin-1 protects PC12 cells against oxidative stress induced by hydrogen peroxide. J. Neurosci. Res..

[57] Zhou Y., Duan S., Zhou Y., Yu S., Wu J., Wu X., Zhao J., Zhao Y. (2015). Sulfiredoxin-1 attenuates oxidative stress via Nrf2/ARE pathway and 2-Cys Prdxs after oxygen-glucose deprivation in astrocytes. J. Mol. Neurosci..

[58] de Franceschi L., Turrini F., Honczarenko M., Ayi K., Rivera A., Fleming M.D., Law T., Mannu F., Kuypers F.A., Bast A. (2004). In vivo reduction of erythrocyte oxidant stress in a murine model of beta-thalassemia. Haematologica.

[59] Hommen F., Bilican S., Vilchez D. (2021). Protein clearance strategies for disease intervention. J. Neural Transm..

[60] Moors T.E., Hoozemans J.J., Ingrassia A., Beccari T., Parnetti L., Chartier-Harlin M.C., van de Berg W.D. (2017). Therapeutic potential of autophagy-enhancing agents in Parkinson’s disease. Mol. Neurodegener..

[61] Turner R.S., Hebron M.L., Lawler A., Mundel E.E., Yusuf N., Starr J.N., Anjum M., Pagan F., Torres-Yaghi Y., Shi W. (2020). Nilotinib Effects on Safety, Tolerability, and Biomarkers in Alzheimer’s Disease. Ann. Neurol..

[62] Simuni T., Fiske B., Merchant K., Coffey C.S., Klingner E., Caspell-Garcia C., Lafontant D.E., Matthews H., Wyse R.K., Brundin P. (2021). Efficacy of Nilotinib in Patients With Moderately Advanced Parkinson Disease: A Randomized Clinical Trial. JAMA Neurol..

[63] Pagan F.L., Wilmarth B., Torres-Yaghi Y., Hebron M.L., Mulki S., Ferrante D., Matar S., Ahn J., Moussa C. (2021). Long-Term Safety and Clinical Effects of Nilotinib in Parkinson’s Disease. Mov. Disord..

[64] Pagan F., Hebron M., Valadez E.H., Torres-Yaghi Y., Huang X., Mills R.R., Wilmarth B.M., Howard H., Dunn C., Carlson A. (2016). Nilotinib Effects in Parkinson’s disease and Dementia with Lewy bodies. J. Parkinsons Dis..

[65] Chou J.L., Wu C.H., Tsai C.Y., Chang A.Y., Chan S.H. (2011). Proteomic investigation of a neural substrate intimately related to brain death. Proteomics.

[66] Dayon L., Turck N., Garci-Berrocoso T., Walter N., Burkhard P.R., Vilalta A., Sahuquillo J., Montaner J., Sanchez J.C. (2011). Brain extracellular fluid protein changes in acute stroke patients. J. Proteome Res..

