# Supplementary material for: Adaptative Up-Regulation of PRX2 and PRX5 Expression Characterizes Brain from a Mouse Model of Chorea-Acanthocytosis
Source: Antioxidants (Basel). 2021 Dec 29;11(1):76. doi: 10.3390/antiox11010076 (PMC8772732; doi:10.3390/antiox11010076)
Supplement: Supplementary file 1 [file antioxidants-11-00076-s001.zip › antioxidants-1513647-supplementary.pdf]

Supplementary Materials

1. Supplementary Methods

1.1 Western-blot analysis. Frozen basal ganglia from each experimental group were homogenized and lysed in iced lysis buffer (HLB, containing 150 mM NaCl, 25 mM bicine, 0.1% SDS, 2% Triton X-100, 1 mM EDTA, protease inhibitor cocktail tablets (Roche), and a final concentration of 1 mM Na_3VO_4), then centrifuged 30 min at 4°C at 12,000 *g* [8, 9]. Packed red cells were lysed in ice-cold phosphate lysis buffer (LB; 5 mM Na_2HPO_4, pH 8.0, containing protease inhibitor cocktail tablets, with final concentrations of 3 mM benzamidine and 1 mM Na_3VO_4) and centrifuged 10 min at 4 °C at 12,000 *g*. Red cell membrane (ghost) and cytosol fractions were obtained as previously reported [8, 9, 19, 34]. Proteins were quantified and analysed by one-dimensional SDS–polyacrylamide gel electrophoresis. Gels were transferred to nitrocellulose membranes for immunoblot analysis with specific antibodies: anti PRX2 (kindly gift of Prof. Chae HZ, Chonnam National University, South Korea), anti PRX5 from AbCam (Cambridge, UK), anti PRX-SO3 (Abcam, Cambridge, UK), anti-Catalase from AbCam (Cambridge, UK), anti GAPDH (clone D6, Santa Cruz Biotechnology, Inc, USA), anti Sulfiredoxin (clone B10, Santa Cruz Biotechnology, Inc, USA), anti-Thioredoxin reductase 1 (clone B2, Santa Cruz Biotechnology, Inc, USA), Phospho Ser40 NRF2 (pNRF2, clone EP1809Y, AbCam, Cambridge, UK), and NRF2 (clone EP1809Y, Abcam, Cambridge, UK), anti NQO-1 (clone C19, Santa Cruz Biotechnology, Inc, USA). Secondary donkey anti-rabbit IgG and anti-mouse IgG HRP conjugated were from GE Healthcare Life Sciences (Little Chalfont, UK). Secondary donkey anti-goat IgG HRP conjugated was from SCBT. Secondary donkey anti-rat IgG HRP conjugated was from AbCam. Blots were developed with Luminata Forte Chemiluminescent HRP Substrate from Millipore (Billerica, MA, USA), and images were acquired with the Image Quant Las Mini 4000 Digital Imaging System (GE Healthcare Life Sciences). Densitometric analyses used ImageQuant TL software (GE Healthcare Life Sciences).

2. Supplementary Results

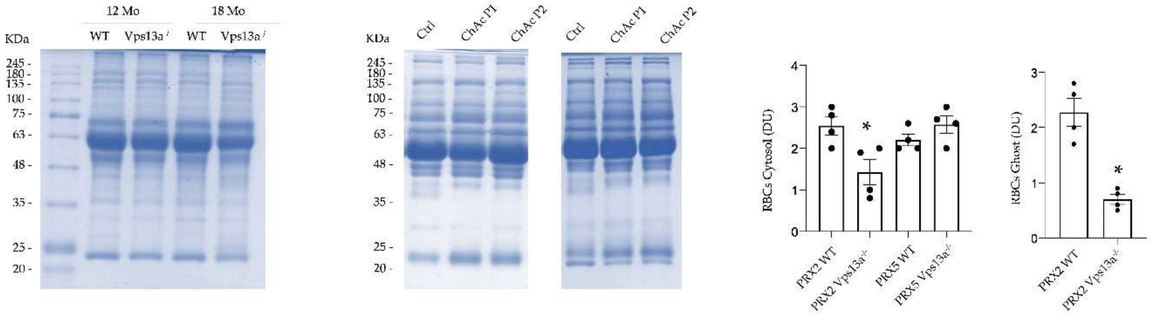

(a) (b) (c)

Figure S1. (**a**) Colloidal Coomassie-stained gels for plasma samples in WT and Vps13a$^{-/-}$ mice at 12 and 18 months (Mo) of age, used as loading control for Figure 1a. Representative of three gels with similar results. (**b**) Colloidal Coomassie-stained gels of plasma samples from healthy control (Ctrl), ChAc patient 1 (P1) and patient ChAc patient 2 (P2), used as loading control for Figure 1b. Representative of three gels with similar results. (**c**) Densitometric analysis of Figure 1d is presented as means ± SEM (n = 4). Data are shown as means ± SEM, n=4; *p<0.05 compared to WT animals.

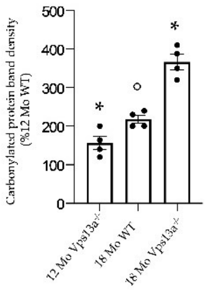

Figure S2. Densitometric analysis of Figure 2a is presented as means ± SEM (n = 4); *p<0.05 compared to WT animals; ° p<0.05 compared to vehicle treated *Vps13a*$^{-/-}$ mice.

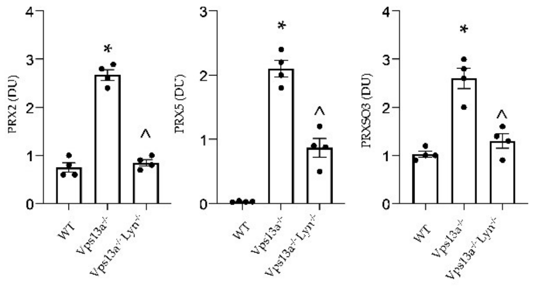

Figure S3. Densitometric analysis of Figure 4a is presented as means ± SEM (n = 4) Data are shown as means ± SEM, n=4; *p<0.05 compared to WT animals; ^ p<0.05 compared to vehicle treated *Vps13a*$^{-/-}$ mice.

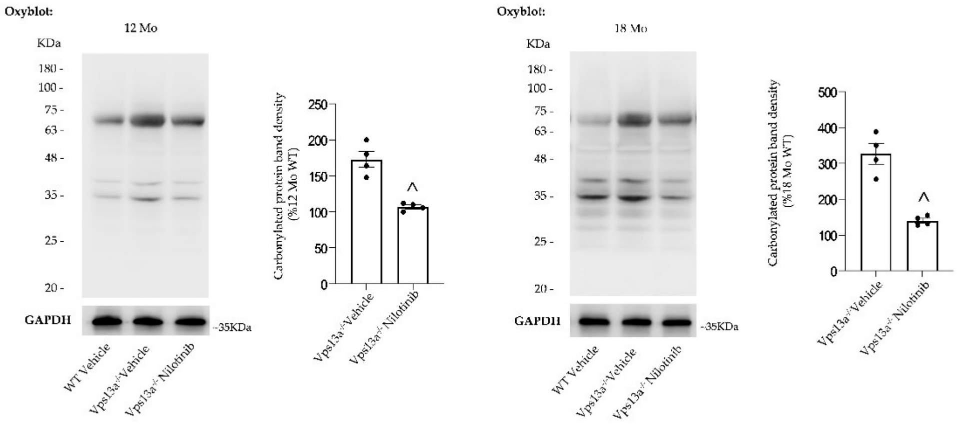

Figure S4. Basal ganglia soluble fraction from WT and *Vps13a*$^{-/-}$ mice at 12 (left panel) and 18 (right panel) months of age treated with either vehicle or nilotinib (25 mg/kg/day for 3 and 6 months). Samples were analyzed on 11% SDS-PAGE and subjected to OxyBlot. The carbonylated proteins (1 mg) were detected by treating with 2,4-dinitrophenylhydrazine and blotted with anti-DNP antibody. Quantification of band area is reported on the right. Data are shown as means ± SEM, n=4; ^ p<0.05 compared to vehicle treated *Vps13a*$^{-/-}$ mice.

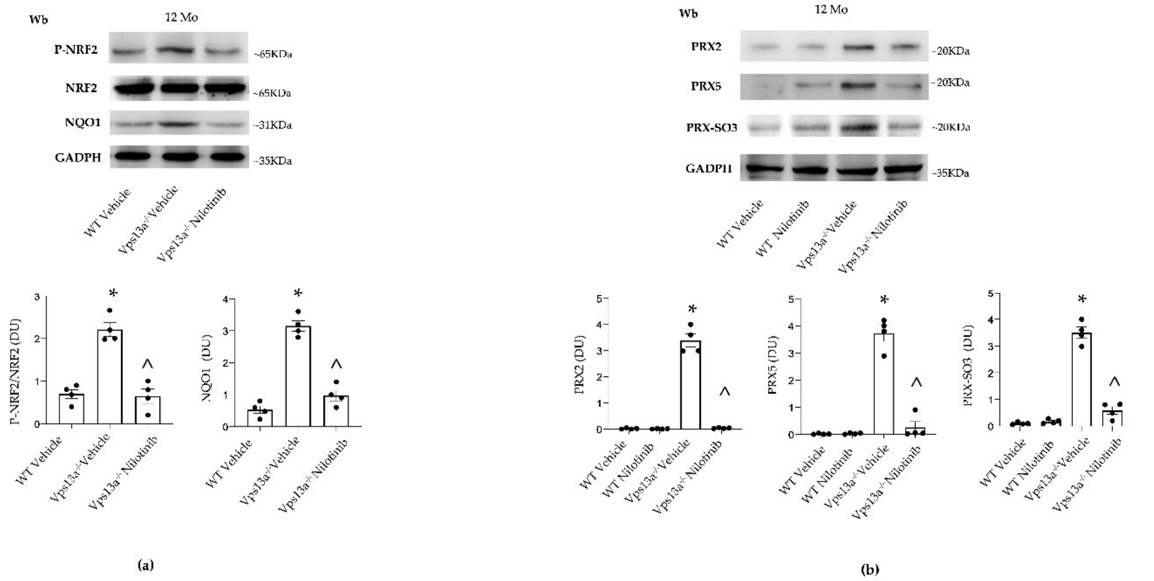

Figure S5. (**a**) Western-blot (Wb) analysis of phospho-NRF2, total NRF2 and NQO1 in isolated basal ganglia of wild-type (WT) and *Vps13a*$^{-/-}$ mice treated with either vehicle or nilotinib (25 mg/kg/day for 3 or 6 months) at 18 months (Mo) of age. GADPH was used as loading control. Representative of 4 blots with similar results. Densitometric analysis is shown in lower panel. Data are shown as means ± SEM, n=4; *p<0.05 compared to WT animals; ^ p<0.05 compared to vehicle treated *Vps13a*$^{-/-}$ mice. (**b**) Western-blot (Wb) analysis of PRX2, PRX5 and Prx-SO3 in isolated basal ganglia of wild-type (WT) and *Vps13a*$^{-/-}$ mice treated with either vehicle or nilotinib (25 mg/kg/day for 6 months) at 18 months of age. GADPH was used as protein loading control. Representative of 4 blots with similar results. Densitometric analysis is shown in lower panel. Data are shown as means ± SEM, n=4; *p<0.05 compared to WT animals; ^ p<0.05 compared to vehicle treated *Vps13a*$^{-/-}$ mice.

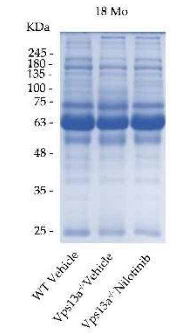

Figure S6. Colloidal Coomassie-stained gels (left panel) for plasma samples in WT and *Vps13a*$^{-/-}$ mice treated with either vehicle or nilotinib (25 mg/kg/day for 6 months) at 18 months of age, used as loading control for Figure 5a. Representative of three gels with similar results.

3. Original images for blots

Figure 1 (a)

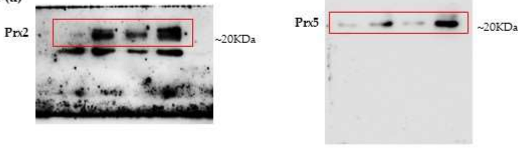

Figure 1 (b)

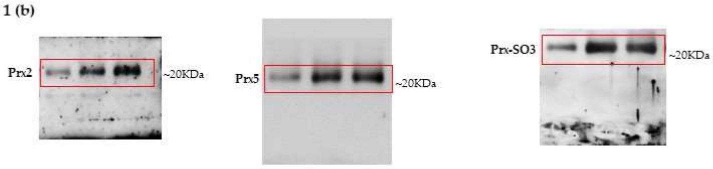

Figure 1 (d)

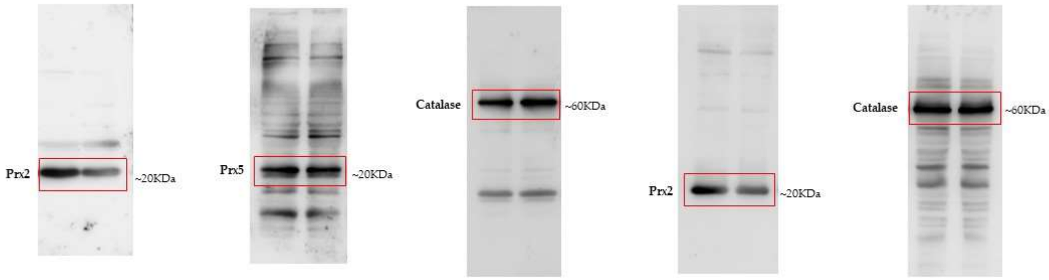

Figure 2 (a)

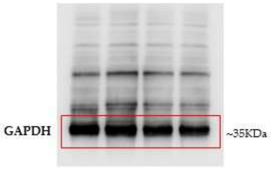

Figure 2 (b)

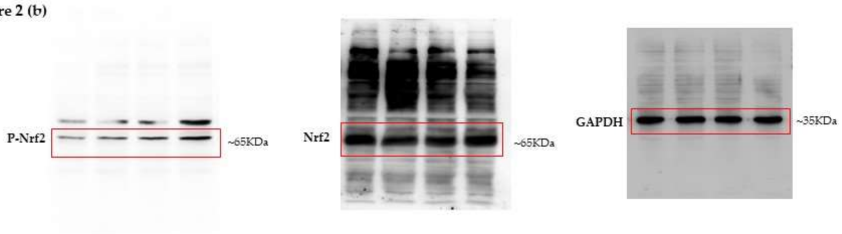

Figure 3 (a)

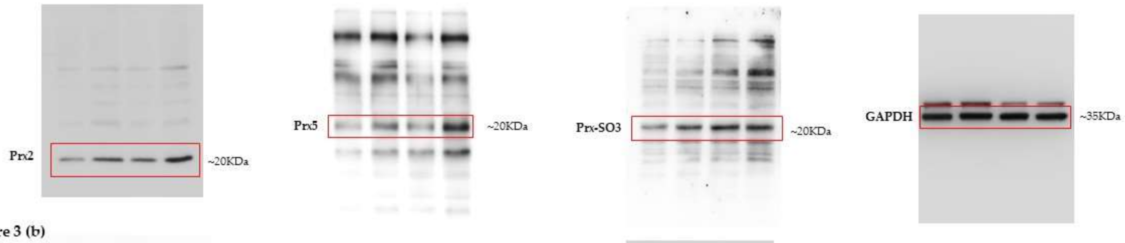

Figure 3 (b)

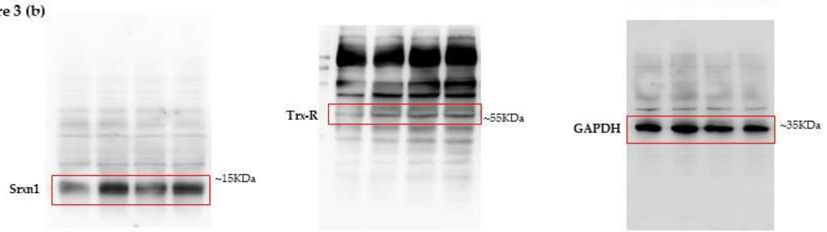

Figure 4 (a)

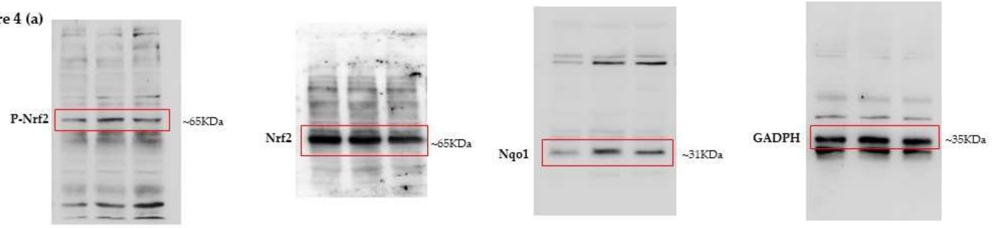

Figure 4 (b)

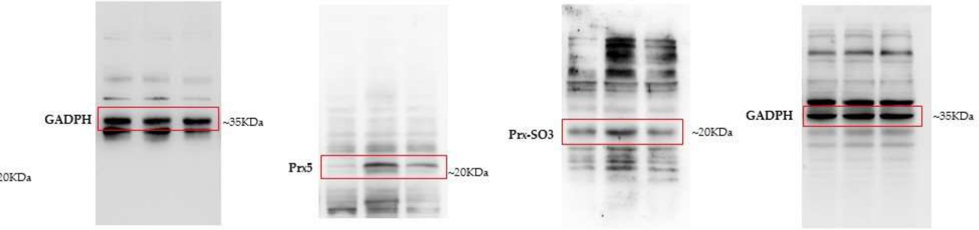

Figure 4 (c)

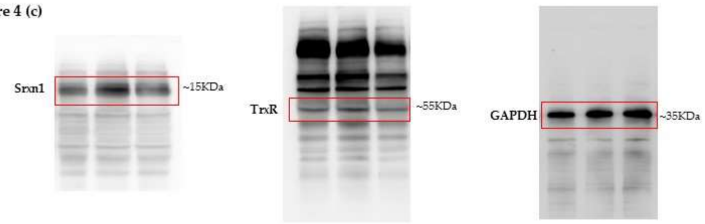

Figure 5 (a)

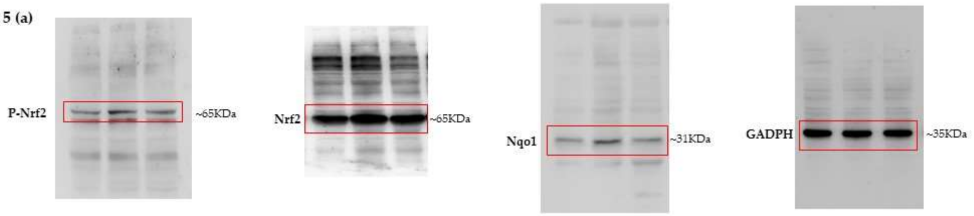

Figure 5 (b)

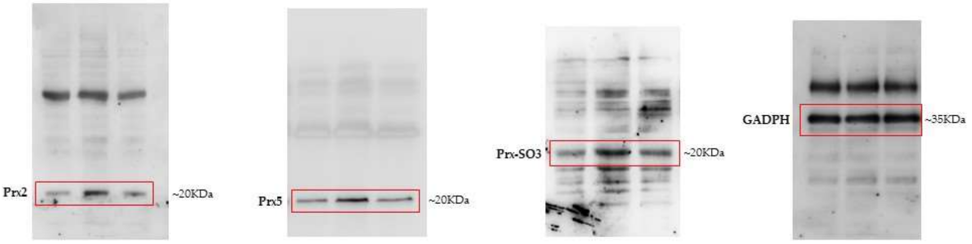

Figure 5 (c)

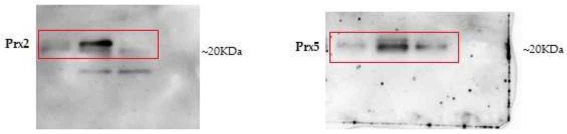

Figure 5S (b)

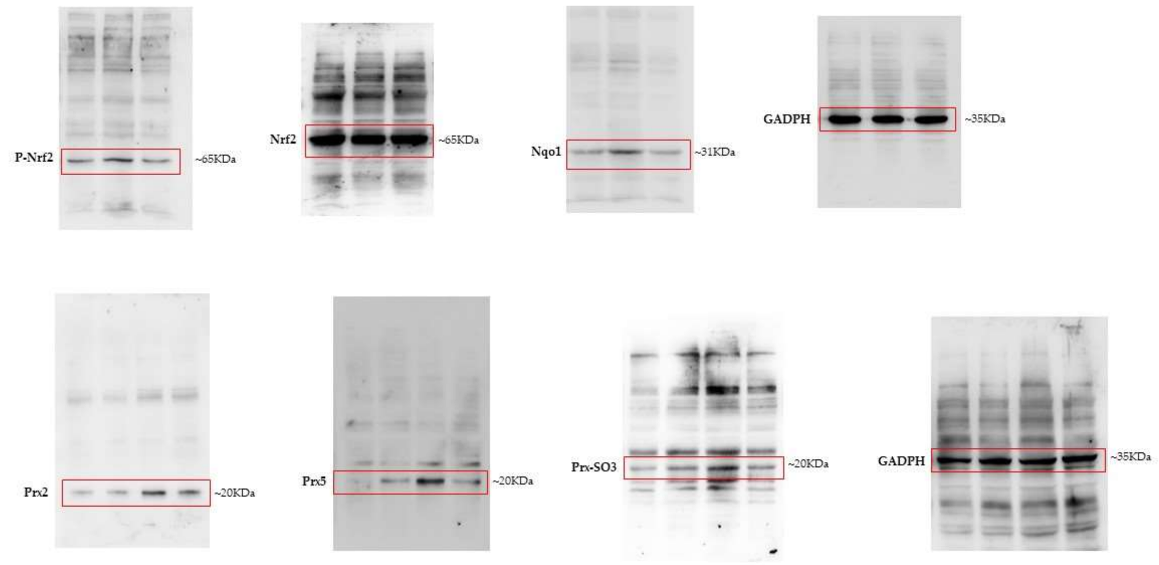
P-Nrf2
~65KDa
Nrf2
~65KDa
Nqo1
~31KDa
GADPH
~35KDa
Prx2
~20KDa
Prx5
~20KDa
Prx-SO3
~20KDa
GADPH
~35KDa
